# Dihydrotestosterone induces SREBP-1 expression and lipogenesis through the phosphoinositide 3-kinase/Akt pathway in HaCaT cells

**DOI:** 10.1186/1476-511X-11-156

**Published:** 2012-11-15

**Authors:** Bing-rong Zhou, Qiu-hong Huang, Yang Xu, Di Wu, Zhi-qiang Yin, Dan Luo

**Affiliations:** 1 Department of Dermatology, First Affiliated Hospital of Nanjing Medical University, Nanjing, 210029, China

**Keywords:** Dihydrotestosterone, SREBP-1, Phosphoinositide 3-kinase/Akt, HaCaT cells

## Abstract

**Background:**

The purpose of this study was to investigate the effects and mechanisms of dihydrotestosterone (DHT)-induced expression of sterol regulatory element binding protein-1 (SREBP-1), and the synthesis and secretion of lipids, in HaCaT cells. HaCaT cells were treated with DHT and either the phosphoinositide 3-kinase inhibitor LY294002 or the extracellular-signal-regulated kinase (ERK) inhibitor PD98059. Real time-PCR, Western blot, Oil Red staining and flow cytometry were employed to examine the mRNA and protein expressions of SREBP-1, the gene transcription of lipid synthesis, and lipid secretion in HaCaT cells.

**Findings:**

We found that DHT upregulated mRNA and protein expressions of SREBP-1. DHT also significantly upregulated the transcription of lipid synthesis-related genes and increased lipid secretion, which can be inhibited by the addition of LY294002.

**Conclusions:**

Collectively, these results indicate that DHT induces SREBP-1 expression and lipogenesis in HaCaT cells via activation of the phosphoinositide 3-kinase/Akt Pathway.

## Introduction

Excessive secretion of sebum on skin is an important factor in various skin diseases, including acne and seborrheic dermatitis [1,2]. Besides sebaceous glands, keratinocytes are another important source of lipid on skin surface (2). Although the epidermis is certainly affected by steroid hormones, little is known about the effects of androgens on human keratinocytes. Previous studies demonstrated that in sebaceous glands androgens regulates the synthesis of sebum lipids through the sterol regulatory element-binding protein (SREBP) pathway [3]. Nevertheless, results of investigations on androgens receptor expression in keratinocytes are controversial: some groups have demonstrated that the androgens receptor is expressed in the epidermis [4], while others reported its absence [5]. Although nothing in the literature so far suggests that testosterone or its analog dihydrotestosterone (DHT) effect the growth of keratinocytes, we found that these hormones were associated with SREBP-1 expression in cells of the immortalized keratinocyte cell line HaCaT, which are quite similar to primary normal keratinocytes in steroid-metabolizing activity and responsiveness to steroid hormones [6].

The SREBP transcription factors bind sterol response elements, and three members of the SREBP family have been identified: SREBP-1a, SREBP-1c, and SREBP-2 [7]. In spite of the partial functional overlap between SREBP-1 and SREBP-2, SREBP-1 typically regulates genes in the fatty acid biosynthesis pathway, whereas SREBP-2 modulates the transcription of genes associated with cholesterol biosynthesis [8]. DHT has been reported to regulates SREBP-1 expression in various cell lines and organs [3,9,10]. Besides that, SREBP-1 was also found to be expressed in keratinocytes and played an important role in its lipid synthesis [11]. However, little is known of how DHT impacts the function of SREBP-1 in keratinocyte HaCaT cells. The aim of this study was to dissect the molecular signaling pathways by which DHT stimulation increases the mRNA and protein levels of SREBP-1 in HaCaT cells.

## Materials and methods

### Materials

Dihydrotestosterone (DHT) powder was bought from Sigma Co. Ltd. DHT was diluted with DMSO just before use, and prepared in serum-free medium at required concentrations, subpackaged by Filtration sterilization. Anti SREBP-1 (polyclonal) was bought from Beijing Bo’ao Bio-tech Co. Ltd.; Rabbit anti phospho-Akt (Ser473) and anti-total Akt was bought from Cell Signaling; Mouse anti phospho-p44/42 MAPK, anti total p44/42 MAPK, Mouse anti phospho-JNK/SAPK, Rabbit anti phospho-P38 MAPK, Mouse/Rabbit anti Actin, PI3K inhibitor LY294002, MEK inhibitor PD98059, BCA Protein Assay Kit were all bought from Beyotime Institute of Biotechnology; Trizol was bought from Invitrogen; Real-time PCR Assay Kits were bought from Nanjing KGI Bioteknologi Development Co, Ltd.; Nile red powder was bought from Shanghai XinRan Bio-tech Co, Ltd, diluted with methanol and stored at a concentration of 10μg/mL at 4°C, away from light.

### Method

#### Cell culture

Keratinocyte line HaCaT cells were cultured in a cell incubator at 37°C, 5% CO_2_, in DMEM medium containing 10% fetal bovine serum and 1% penicillin and streptomycin. After cells became polygon arranging as a single layer, they were vaccinated at the density of 1*10 ^9^/L with 0.25% trypsin solution. The cultured cells were used for experiment when they adhered to the culture plat and the confluence reached 70%~80%.

#### Experiment grouping and treatment of cells

To examine the effect of DHT on the expression of SREBP-1 and the activation of the phosphoinositide 3-kinase (PI3k)/Akt and mitogen-activated protein kinase (MAPK) pathways in HaCaT cells, the cells were first randomly divided into 4 groups based on DHT treatment concentrations: 0 (control), 10, 100, and 1000 nM. DHT at a concentration of 100 nmol/L was taken as the stimulus quantity, and random groups were created: $\bar{\text{a}}$ control group; α a DHT group at a concentration of 100nmol/L; βPD98059 group, pretreated with PD98059 at 50μM for 40 min; χ LY294002 group, pre-treated with LY294002 at 50μM for 40min; δPD98059+DHT group, pretreated with PD98059 at 50μM for 40 min, then with DHT added at concentration of 100 nmol/L; ε LY294002+DHT group, pre-treated with LY294002 at 50μM for 40min, then with DHT added at concentration of 100nmol/L. Every group was cultured for 24 hours before further tests.

#### Real-time PCR detection of the expression of SREBP-1 mRNA and the expression of lipogenic enzyme (FAS, ACS, SCD, HMGCR) mRNA in HaCaT cells

Cells were grouped according to experimental grouping requirements. Trizol was added to break down the cells, followed by extraction of total RNA, measurement of concentration and then measurement of purity. After ensuring that the quality met the requirements of the experiment, cDNA was obtained by reverse transcription. It was diluted 10 times and amplified according to a 20μL reaction system. Primers were synthesized by Nanjing Kaiji Bio-tech Co, Ltd (Additional file 1 Table S1). Amplification conditions: pre-degeneration at 95°C for 5 min, entering reaction circles, degeneration at 95°C for 15 min, annealing for 30s at 60°C, extending for 30s at 72°C, keeping at 72°C for 10 min after 40 circles.

#### Western blotting tests for the protein expression of SREBP-1, p-AKT, p-P38 and p-JNK

The cells were treated according to experiment grouping methods, the culture solution was discarded, the cells were washed 3 times with PBS solution pre-cooled to 4°C, 300ul cell degradation solution containing protease inhibitor was added, the mixture was placed on ice for 15min, cells were detached and centrifugal separation was conducted at 4°C at 12000rpm. The upper layer solution was taken for protein testing by the BCA method. A 30μg sample of protein was taken from each group for SDS-PAGE electrophoresis testing, and protein was transferred onto PVDF films, covered with a 5% BSA blocking buffer at 37°C for 1 hour. The primary antibody was added according to the experiment requirements (Rabbit antibody SREBP-1, dilutedat 1:500, Rabbit antibody phospho-AKT, diluted at 1:1000, Rabbit antibody total-AKT, diluted at 1:1000; mouse antibody phospho-ERK, diluted at 1:1000, mouse antibody total-ERK, diluted at 1:1000; Rabbit antibody phospho-P38, diluted at 1:1000; mouse antibody phospho-JNK, diluted at 1:1000; Actin antibody diluted at 1:1000), kept in incubation at 4°C throughout the night, washed before being incubated with the secondary antibody, which was diluted at 1:1000 and marked by horseradish peroxidase, at 37°C for 40 min. ECL detection reagent was added for 5min, and finally squash, development and fixation were conducted.

#### Observing the DHT-induced lipid droplet changes in HaCaT cells by the Oil Red staining method

HaCaT cells were inoculated at exponential phase in 12-well plates, 3×10^4^ for each well, and cultured for 24 hours. HaCaT cells were treated according to experiment grouping methods, and after the culture medium solution was carefully discarded were washed with PBS solution 3 times, then fixed with 4% paraformaldehyde for 10min. The cells were colored with Oil Red staining solution for 20min,washed with 60% isopropanol solution for 30s, washed with distilled water for 30s until the background was clear, and then the culture plates were placed upside down under a microscope and photographed with Leica Qwin Plus (plain light).

#### Detecting the synthesis of lipid in HaCaT cells by flow cytometer

HaCaT cells at exponential phase of growth were inoculated in 6-well plates, 3×10^4^ for each well, and cultured for 24 hours. The HaCaT cells were treated according to experiment grouping methods, and 0.25% trypsin solution containing 0.02%EDTA was added. The degradation process was ended with10% fetal calf serum medium. The cells were washed 2 times with PBS, single cell suspension was prepared (in PBS), 100ng/mL nile red fluorescent dye was added, samples were incubated at room temperature for 15min, filtered with 300 mesh nylon membrane, and then flow cytometry was used to test 10000 cells of each sample . The average fluorescence intensity of every cell was calculated, with excitation wavelength at 485nm and emission wavelength at 565nm, and 3 well plates in each group.

### Statistical analysis

SPSS13.0 software was used for data analysis, and the form of average±standard deviation $\left(\bar{\text{x}}\text{+s}\right)$ was used to indicate measurement data. ANOVA was used for inter-group comparison, P<0.05 was considered statistically significant.

## Results and discussion

**Figure 1:**
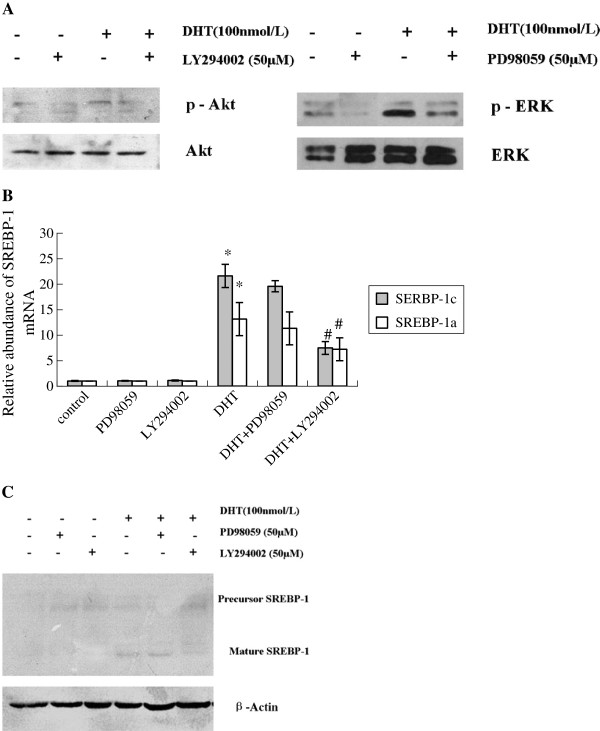
**The effects of the PI3K inhibitor (LY294002) and ERK inhibitor (PD98059) on DHT -induced expression of SREBP**-**1 in HaCaT cells.** (**A**) LY294002 and PD98059 distinctly inhibited the DHT-induced protein expressions of p-Akt and p-ERK, respectively. (**B**) LY294002 inhibited the DHT-induced expression of SREBP-1 mRNA, while PD98059 has no significant effect on the DHT-induced mRNA expression of SREBP-1. (**C**) LY294002 inhibited the DHT-induced protein expression of SREBP-1, while PD98059 has no significant effect on the DHT-induced expression of protein SREBP-1. Data are means±SD of three independent experiments. * *P* < 0.01 vs. untreated control group; ^#^*P* < 0.05 vs. DHT group.

**Figure 2:**
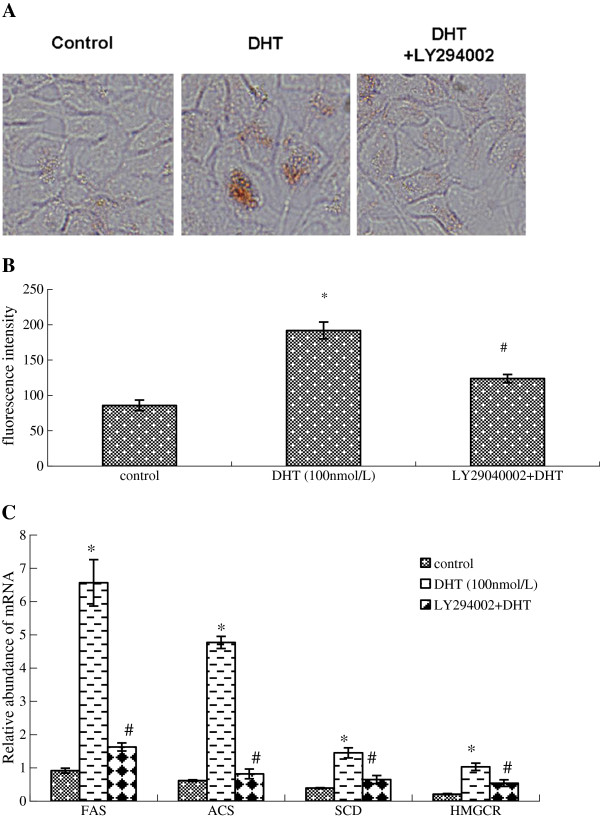
**The effect of LY294002 on DHT**-**induced expression of lipid synthetase**-**related genes and lipid synthesis in HaCaT cells**. (**A**) The effect of LY294002 on the synthesis of lipids, as shown using Oil Red staining. (**B**) The effect of LY294002 on the synthesis of lipids, as tested using flow cytometry. (**C**) The effect of LY294002 on DHT-induced expression of lipid synthetase related genes, as tested by real-time PCR. Data are means±SD of three independent experiments. * *P* < 0.01 vs. untreated control group; ^#^*P* < 0.05 vs. DHT group.

DHT treatment increased SREBP-1a and SREBP-1c mRNA levels and SREBP-1 protein expression in HaCaT cells in a dose-dependent manner (Additional file 2 Figure S1a-b). p-AKT in the PI3k/Akt pathway and p-ERK pathway were also significantly upregulated by DHT in a dose-dependent manner. However, DHT has no effect on the p-P38 and p-JNK expression in the MAPK pathways (Additional file 2 Figure S1-C). The PI3k/Akt inhibitor LY294002 and the ERK inhibitor PD98059 could reverse the activation of p-Akt and p-ERK (Figure 1-A), respectively, and their effects on DHT-induced SREBP-1c expression were different: LY294002 inhibited the DHT-induced activation of SREBP-1, but PD98059 had little effect (Figure 1, B, C). Compared with the control group, DHT stimulated HaCaT cells to synthesize more lipid droplets. After pretreatment with LY294002, the droplets were significantly abolished (Figure 2a), which was consistent with the results from flow cytometry analysis of Nile Red fluorescent dye intensity (Figure 2b). FAS, ACS, SCD, and HMGCR are all important enzymes with a role in lipid synthesis, whose gene transcription is regulated by SREBP-1 [12]. When DHT was introduced, the mRNA levels of FAS, ACS, SCD, and HMGCR were significantly increased. However, the addition of LY294002 disrupted these DHT-mediated effects (Figure 2c).

Abundant secretion of lipid in epidermis and sebaceous glands caused by androgen contributes in a major way to the occurrence of diseases like acne and seborrheic dermatitis. The process of lipid synthesis involves the activation of lipid synthesis related enzyme genes in combination with regulatory factors. SREBPs were discovered to be the major nuclear transcription factors contributing to the regulation of lipid synthesis and secretion, being able to activate the transcription process of such enzyme genes as FAS, ACS, SCD, HMGCR, and in this way stimulating lipid synthesis and secretion [11,13-15]. In recent years, much attention has been focused on intracellular signal transduction mediating the activation of the SREBP pathway, which plays a role in lipogenesis. The PI3K/Akt signaling and MAPK pathways both play important roles in mediating the activation of the SREBP pathway [16]. With differences in tissue and cell types and different stimulation factors there are differences in the signaling pathways which activate the SREBP pathway. Yang YA et al. [17] found that P13k/Akt and ERK/MAPK signaling pathways may play a role in regulation of the expression of SREBP-1 and the transcription of FAS in breast cancer. KGF can induce the expression of SREBP-1 in H292 lung cancer cells through two pathways (JNK and PI3K/Akt), so enhancing the expression of SCD-1 and FAS [18]. Smith TM et al. [12] studied the effects of PI3k/Akt and MAPK pathways on the IGF-1-induced expression of SREBP-1 in sebocytes and found that PI3k/Akt played an important role in this process. Our results shows that whether the PI3k/Akt pathway is activated or not has a close bearing on the expression of SREBP-1 induced by DHT, which may be important and specific in DHT treated keratinocyte HaCaT cells.

Recent reports indicate that activation of Akt is involved in the transport of the SREBP cleavage-activating protein (SCAP)/SREBP complex from the endoplasmic reticulum to the Golgi [19]. This is a major regulatory step in SREBP activity. Our data indicate that DHT increases the amount of cleaved (mature) SREBP protein and that this increase is inhibited in the presence of LY294002 (Figure 1b,c). This suggests that, in addition to possible transcriptional and translational control, Akt activation may also affect SREBP processing in HaCaT cells. Akt activation has been shown to increase the expression of lipogenic genes [16]. The conclusion is further supported by our observation that inhibition of Akt activation by LY294002 blocks the increase in mRNA expression of lipogenic genes and lipogenesis induced by SREBP-1 (Figure 2c).

## Conclusions

In conclusion, our investigations suggest that DHT can activate SREBP-1 and lipogenesis in HaCaT cells and that the PI3k/Akt signaling pathway has important regulatory effects on lipid synthesis and secretion induced by DHT.

## Abbreviations

ACS: Acyl-Coa Synthetase; AKT: v-Akt murine Thymoma viral oncogene homolog; DHT: DiHydroTestosterone; ERK: Extracellular-signal-Regulated Kinase; FAS: Fatty Acid Synthase; HMGCR: HMG-CoA reductase; MAPK: Mitogen-Activated Protein Kinase; p-ERK: Phosphorylated ERK; p-AKT: Phosphorylated AKT; PI3K: Phosphoinositide 3-kinase; SCAP: SREBP cleavage-activating protein; SCD: Stearoyl-Coa Desaturase; SREBP-1: Sterol Regulatory Element Binding Protein-1.

## Competing interests

The authors declare that they have no competing interests.

## Authors’ contributions

BRZ conceived, designed and coordinated the work, as well as prepared the manuscript. DL was involved in the co-design of the work as well as the draft of the manuscript. HQH, YX, DW and ZQY carried out analytical work and contributed in drafting the manuscript. All authors read and approved the final manuscript.

## Authors’ information

BRZ is a medical scientific researcher. YX, DW and ZQY are Ph.D. students; HQH is a M.Sc. student; DL is a full professor of dermatology.

## Supplementary Material

Additional file 1 **Table S1.** Primers and amplified products. Click here for file

Additional file 2 **Figure S1.** The effects of DHT on the expressions of SREBP-1, p-P38 and p-JNK in HaCaT cells. Click here for file
