# Supplementary material for: Dihydrotestosterone induces SREBP-1 expression and lipogenesis through the phosphoinositide 3-kinase/Akt pathway in HaCaT cells
Source: Lipids Health Dis. 2012 Nov 15;11:156. doi: 10.1186/1476-511X-11-156 (PMC3528431; doi:10.1186/1476-511X-11-156)
Supplement: Additional file 1 — Table S1. Primers and amplified products. [file 1476-511X-11-156-S1.doc]

Table s1. Primers and amplified products

| Genes | Primers | Products |
| --- | --- | --- |
| SREBP-1a | Forward primer:  5' GTG ACC GAT GGC TTC AGT TCC CTG 3'  Reverse primer:  5' GGt AGG AGA GCA CTC AGA ATA CA 3' | 233 bp |
| SREBP-1c | Forward primer:  5' GGAGCCATGGATTGCACTTT 3'  Reverse primer:  5' TCAAATAGGCCAGGGAAGTCA 3' | 77 bp |
| FAS | Forward primer:  5' CAGGCACACACGATGGAC 3'  Reverse primer:  5' CGGAGTGAATCTGGGTTGAT 3' | 92 bp |
| ACS | Forward primer:  5' CCCAGTTTATCCCAATGCTG 3'  Reverse primer:  5' GGGCGCCATAGAACTGATT 3' | 74 bp |
| SCD | Forward primer:  5' CCGGGAGAATATCCTGGTTT 3'  Reverse primer:  5' GCGGTACTCACTGGCAGAGT 3' | 97 bp |
| HMGCR | Forward primer:  5' TGGCTCTTTCAGAGAGGTCTCA 3'  Reverse primer:  5' TGCCTTCAGAGGTGAGCTGTA 3' | 158 bp |
| Actin | Forward primer:  5' GCAGAAGGAGATCACAGCCCT 3'  Reverse primer:  5' GCTGATCCACATCTGCTGGAA3' | 136 bp |
