# Supplementary material for: Dihydrotestosterone induces SREBP-1 expression and lipogenesis through the phosphoinositide 3-kinase/Akt pathway in HaCaT cells
Source: Lipids Health Dis. 2012 Nov 15;11:156. doi: 10.1186/1476-511X-11-156 (PMC3528431; doi:10.1186/1476-511X-11-156)
Supplement: Additional file 2 — Figure S1. The effects of DHT on the expressions of SREBP-1, p-P38 and p-JNK in HaCaT cells. [file 1476-511X-11-156-S2.doc]

**Supplementary Figure Legend**

**A**

**B**


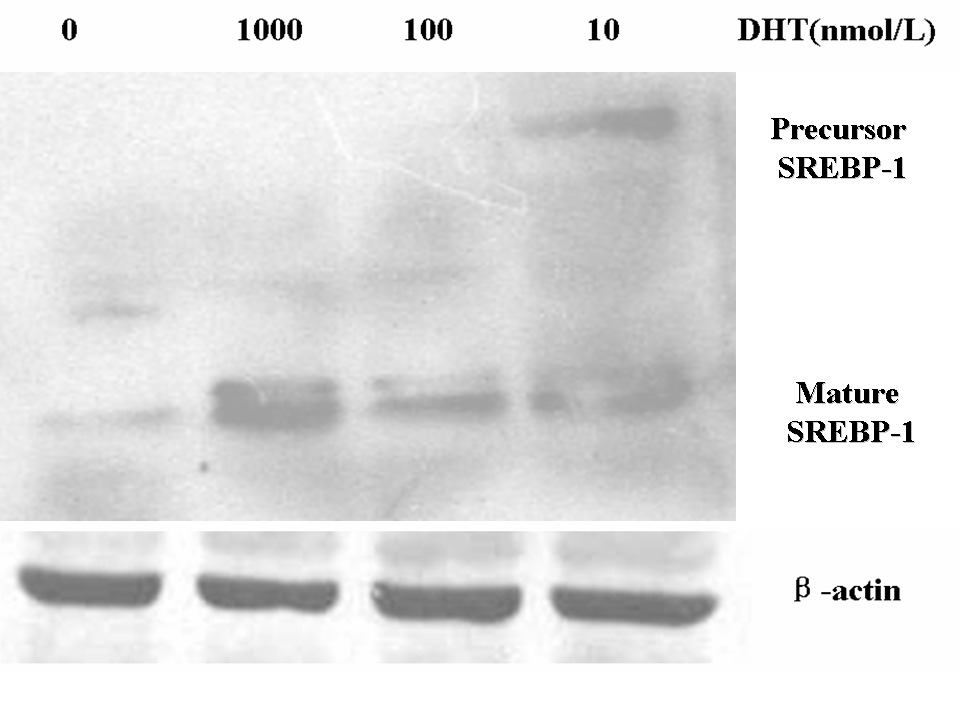


**C**


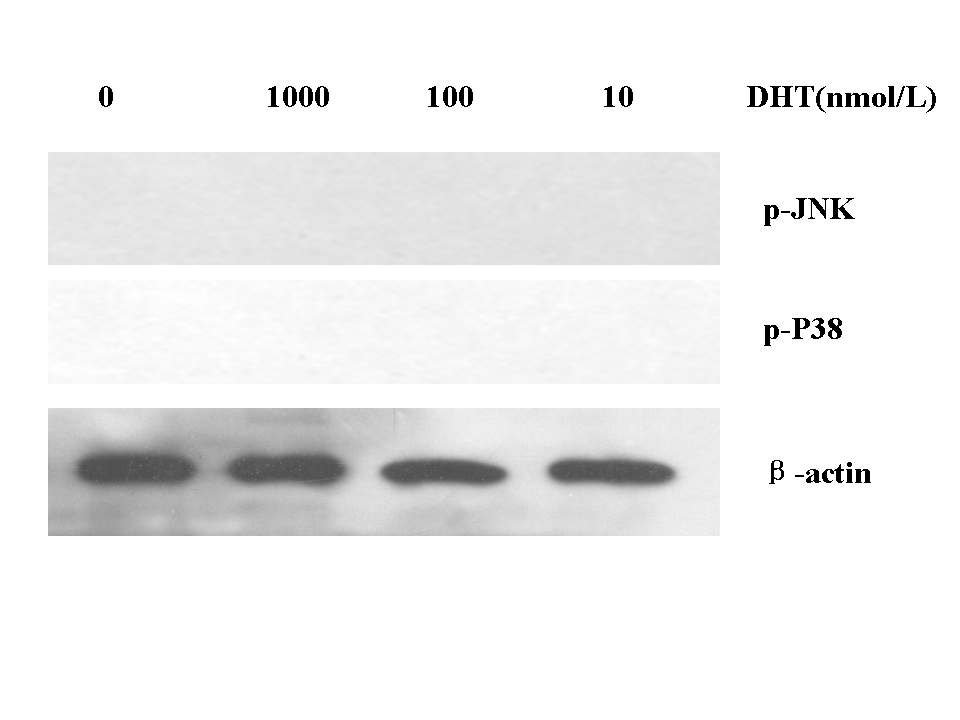


**Figure S1.** The effects of DHT on the expressions of SREBP-1, p-P38 and p-JNK in HaCaT cells. (A) DHT increases the mRNA expression of SREBP-1c in a dose-dependent manner. (B) DHT increases the protein expression of SREBP-1 in a dose-dependent manner. (C) DHT has no significant impact on p-P38 and p-JNK protein expression in HaCaT cells. **P* < 0.01 vs. blank control group.
